# Uric acid induces stress resistance and extends the life span through activating the stress response factor DAF-16/FOXO and SKN-1/NRF2

**DOI:** 10.18632/aging.102781

**Published:** 2020-02-12

**Authors:** Qin-Li Wan, Xiaodie Fu, Wenyu Dai, Jing Yang, Zhenhuan Luo, Xiao Meng, Xiao Liu, Ruowei Zhong, Hengwen Yang, Qinghua Zhou

**Affiliations:** 1 Zhuhai Precision Medical Center, Zhuhai People’s Hospital (Zhuhai Hospital Affiliated with Jinan University), Jinan University, Guangdong 510632, Guangzhou, China; 2 The Biomedical Translational Research Institute, Faculty of Medical Science, Jinan University, Guangdong 510632, Guangzhou, China; 3 Qingyuan People's Hospital, The Six Affiliated Hospital of Guangzhou Medical University, Guangdong 511518, Qingyuan, China; 4 Internship Program, The Biomedical Translational Research Institute, Faculty of Medical Science, Jinan University, Guangdong, 510632, Guangzhou, China

**Keywords:** *Caenorhabditis elegans*, longevity, uric acid, DAF-16/FOXO, SKN-1/NRF2

## Abstract

Uric acid is a common metabolite found in mammals’ serum. Recently, several metabolites have been identified that modulate aging, and uric acid levels are positively correlated with mammals’ lifespan. However, the molecular mechanisms underlying this are largely undefined. Here we show that uric acid, an end product of purine metabolism, enhances the resistance of oxidative stress and extends the life span of *Caenorhabditis elegans (C. elegans)*. We show that uric acid enhances a variety of pathways and leads to the upregulation of genes that are required for uric acid-mediated life span extension. We find that the transcription factors DAF-16/FOXO, SKN-1/NRF2 and HSF-1 contribute to the beneficial longevity conferred by uric acid. We also show that uric acid induced life span extension by regulating the reproductive signaling and insulin/IGF-1 signaling (IIS) pathways. In addition, we find that mitochondrial function plays an important role in uric acid-mediated life span extension. Taken together, these data suggest that uric acid prolongs the life span of *C. elegans*, in part, because of its antioxidative activity, which in turn regulates the IIS and the reproductive signaling pathways, thereby activating the function of the transcription factors DAF-16, HSF-1 and SKN-1.

## INTRODUCTION

The process of aging has fascinated humankind for thousands of years. Aging has been defined as a synchronous global decline in physiological and psychological function, accompanied by many diseases, including type 2 diabetes, cancer and hypertension [1, 2]. One of the main mechanisms underlying aging and age-associated disease is a chronic elevation of reactive oxygen species (ROS) [3]. It has been nearly 50 years since Harman proposed the “free radical theory” of aging [4]. The initial theory suggested that, under the stimulation of the external environment, the body will continue to produce ROS. In addition, several important defense mechanisms in the body (i.e., ROS scavengers, protein repair, refold machinery and molecular degradation apparatuses) are also regulated to maintain ROS homeostasis in the body. However, when these defense mechanisms are compromised, ROS levels are elevated to excess and are responsible for the processing of aging [5, 6]. The classic “free radical theory” has been broadly accepted as an explanation for aging, and several lines of evidence previously reported suggested that the reduction of ROS could ameliorate aging and age-related diseases in a wild spectrum of model organisms [7, 8]. In addition, the oxidative stress theory is the theoretical backbone for the use of antioxidants as antiaging supplements, including vitamin C and N-acetyl cysteine (NAC). In addition to these two compounds, some antioxidants that can affect life span and health span have been discovered, such as melatonin [9], hydralazine [10], cytoprotective polyphenol [11], peptides [12] and polysaccharides [13], which help alleviate oxidative stress and delay aging.

Uric acid, as the end product of purine metabolism in the human body, is one of the most abundant antioxidant molecules that can scavenge peroxynitrite and hydroxyl radicals to prevent lipid peroxidation [14, 15]. Studies in animal models have shown that the administration of uric acid or uric acid analogs could protect the brain against ischemic injury due to its antioxidant properties [16, 17]. In addition, a previous study showed that a uric acid analog used to accelerate wound healing also could protect cells against injury [18]. These findings from epidemiological, clinical and experimental studies suggest that uric acid benefits health to some extent. However, whether uric acid plays a role in aging and the basis of such a function remains poorly characterized. There is only one recent study showing that uric acid could enhance longevity and protect the brain against ischemia in mice [19]. However, there has not been an in-depth investigation into the mechanism of uric acid conferred benefits to longevity in a published study.

In this study, we used *Caenorhabditis elegans (C. elegans)* as a model organism to explore the molecular mechanism of delaying aging induced by uric acid. The free-living soil nematode *C. elegans* has become a powerful model organism for studying the molecular mechanisms of aging due to its short life span and ease of genetic manipulation, and most importantly, *C. elegans* has evolutionally conserved longevity and stress genes [20]. Our results from this study indicated that uric acid significantly extended the life span, delayed age-related physiological functions, and enhanced oxidative stress resistance in *C. elegans* by activating the stress-related transcription factors DAF-16/FOXO and SKN-1/NRF2 and by regulating the insulin/IGF-1 signaling (IIS) and reproductive signaling pathways.

## RESULTS

### Uric acid extends the *C. elegans* life span and health span

**Figure 1:**
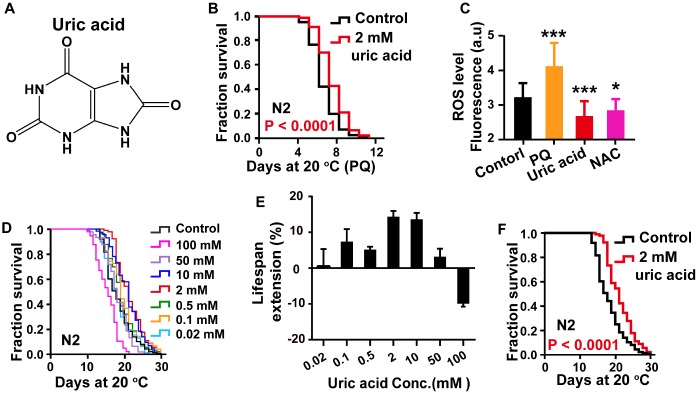
**Uric acid extends the life span of *C. elegans*.** (**A**) Chemical structure of uric acid. (**B**) Survival of animals treated with 2 mM uric acid on paraquat and the untreated controls. (**C**) Quantitation of intracellular levels of ROS in animals treated with 2 mM uric acid and those in untreated controls at day 1 adulthood. PQ is the abbreviation for paraquat, and NAC represents N-acetyl-cysteine. Data are the means ± SD, n ≥ 30, * P < 0.05, *** P < 0.001 (Student’s t test). (**D**) Survival analysis of wild-type N2 animals raised at increasing concentrations (0.02-100 mM). (**E**) Dose-response analysis of uric acid. The average life span changes from at least three independent experiments. (**F**) Survival curves of animals treated with 2 mM uric acid at 20 °C and those of untreated controls. Uric acid exposure was administered beginning on day 1 of adulthood. Life span was analyzed using the Kaplan-Meier test, and P values were calculated using the log-rank test. Data are representative of at least three independent experiments, and details on the life span values are summarized in Supplementary Table 1 in the Supporting Information.

To confirm the antioxidant activity of uric acid (Figure 1A), we first examined the response of uric acid to oxidative stress using a paraquat resistance assay in wild-type *C. elegans*. The survival rate of worms treated with different concentration (0.02 mM, 0.1 mM, 0.5 mM and 2 mM) of uric acid was determined under hyperoxia induced by 5 mM paraquat. We found that treatment with 2 mM uric acid significantly protected animals against oxidative stress, indicating that uric acid has antioxidant activity (Figure 1B, Supplementary Figure 1, Supporting information).

Additionally, when the stimuli are generated by the external environment, the body continuously produces ROS, which are responsible for the process of aging, according to the free-radical theory of aging [3, 21]. To further analyze the antioxidant action of uric acid in vivo, the intracellular ROS accumulation levels were measured by using 2′,7′-dichlorodihydro-fluorescein diacetate (H2DCF-DA), a free radical sensor, which is deacetylated by intracellular esterases, emitting fluorescence signals that correlate with intracellular ROS levels [22]. Our results showed that uric acid treatment significantly decreased the level of ROS compared with the nontreatment control condition (Figure 1C).

Antioxidant activity is known to have longevity-promoting effects in various organisms [23, 24]. To assess whether the antioxidant activity of uric acid is associated with a prolonged life span, we analyzed the effect of uric acid on the life span of wild-type N2 worms. Recently, it is reported that high concentrations of uric acid reduces life span of *drosophila* [25]. Therefore, we examined the effect of higher concentration of uric acid on life span of *C. elegans* in addition to the concentration used in the paraquat resistance experiment. Our results showed that uric acid extended the life span of wild-type *C. elegans* in a dose-dependent manner (Figure 1D–1F). Uric acid was most effective at 2 mM when administered to *C. elegans* in adult stages, and the mean life span increased by 14.68% compared with that of the untreated control. Consistent with recent reported in *drosophila*, life span of *C. elegans* was significantly shortened when animals treated with higher concentration of uric acid (100 mM). Therefore, we treated *C. elegans* with 2 mM uric acid in the subsequent experiments. Accordingly, these results demonstrated that uric acid promoted longevity because of its antioxidant activity.

**Figure 2:**
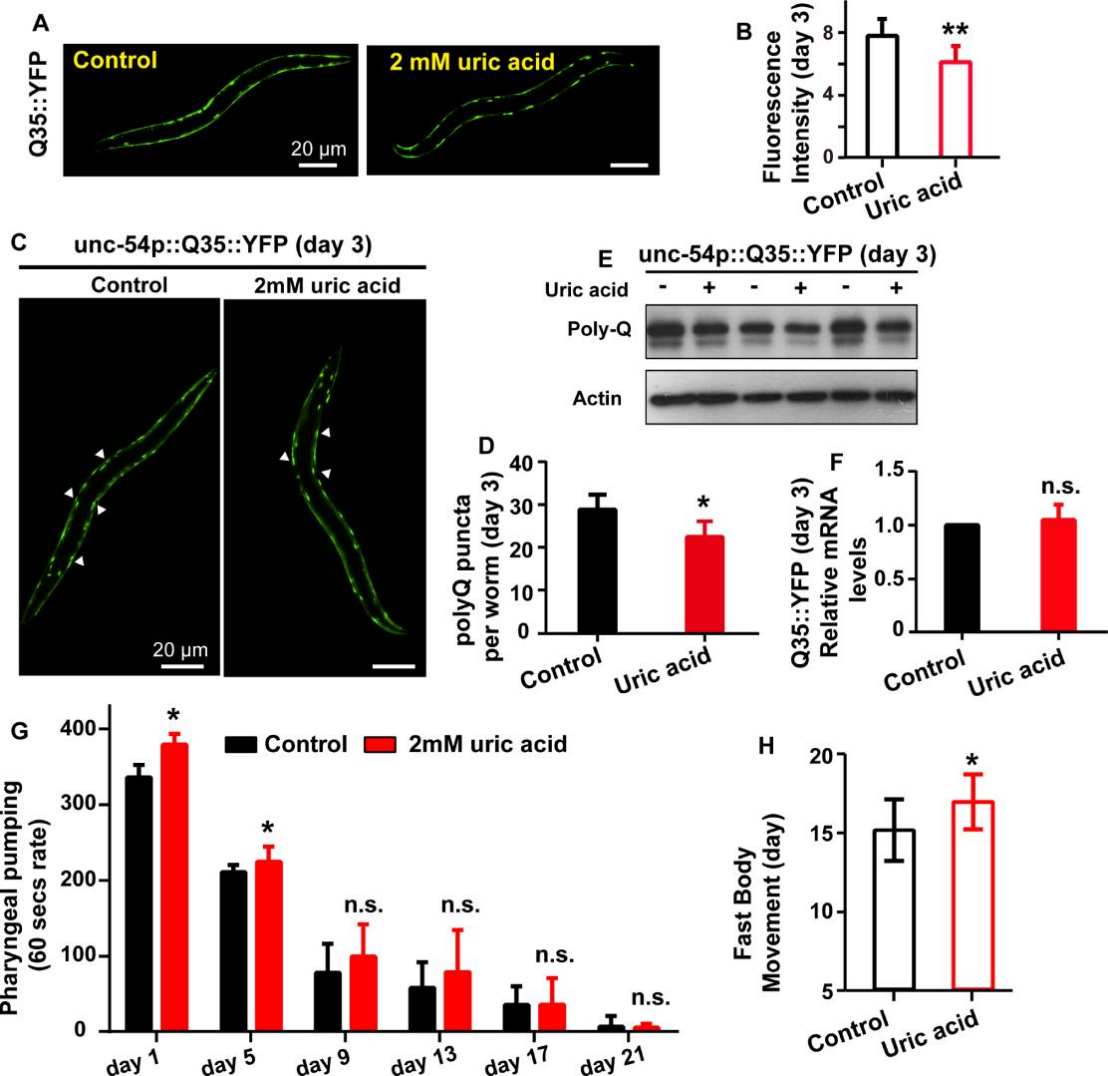
**Uric acid extends the health span of *C. elegans*.** (**A** and **B**) Image and quantitation of the fluorescent intensity of AM140 on day 3 of adulthood. Data are the means ± SD, n ≥ 30, ** P < 0.01 (Student’s t test). (**C** and **D**) Image and quantitation of the polyQ puncta aggregates of AM140 on day 3 of adulthood. Data are the means ± SD, n ≥ 30, * P < 0.05 (Student’s t test). (**E**) Western blot analysis of polyQ35::YFP in the presence and absence of 2 mM uric acid at day 3 of adulthood. (**F**) mRNA level of Q35::YFP when animals treated with or without 2 mM uric acid on day 3 of adulthood (means ± SD, n = 3, n.s.: no significant difference, Student’s t test). (**G**) Effects of 2 mM uric acid on pharyngeal pumping. * P < 0.05 for uric acid versus control at day 1 and day 5 adulthood. Data are the means ± SD, n = 20-30 animals (there were fewer animals on later days), Student’s t test. (**H**) Effects of 2 mM uric acid on period of fast movement (means ± SD, n ≥ 60, * P < 0.05, Student’s t test).

To investigate whether the uric acid-mediated life span extension is related to extend life span and enhance health span, we measured the effect of uric acid on the fluorescence intensity and puncta aggregation of polyglutamine (polyQ) AM140 (Punc-54::Q35::YFP) fused to the yellow fluorescent protein (YFP) and expressed in a transgenic strain. The compound is found in the body wall muscle, and the signal indicates age-dependent polyQ aggregation. We found that *C. elegans* treated with 2 mM uric acid had significantly reduced age-related polyQ aggregation at day 3 (Figure 2A–2D) and day 4 (Supplementary Figure 2A–2D) during adulthood compared with that of the untreated controls. Consistently, the protein level of polyQ were also reduced when animals treated with 2 mM uric acid at day 3 (Figure 2E) and day 4 (Supplementary Figure 2F). However, no reduction in polyQ transcript levels was observed when animals supplement with 2 mM uric acid at day 3 (Figure 2F) and day 4 (Supplementary Figure 2E), suggesting that polyQ protein level reduction by uric acid was likely to be mediated through affecting the protein clearance.

Aging is accompanied by the decline of many phenotypes including body movement and pharyngeal pumping. We detected the effect of uric acid on pharyngeal pumping and period of fast movement. Our results exhibited that period of fast movement was extended when animals treated with uric acid, suggesting that the decrease in body movement with aging was delayed by uric acid (Figure 2H). However, no significant difference in pharyngeal pumping rate was observed, except pharyngeal pumping rate displayed moderately increased at day 1 and day 5 adulthood (Figure 2G). According to the above results, we concluded that uric acid significantly prolonged youthfulness and increased the health span of *C. elegans* because of its antioxidant activity.

### Uric acid extends the life span by activating SKN-1/NRF2

**Figure 3:**
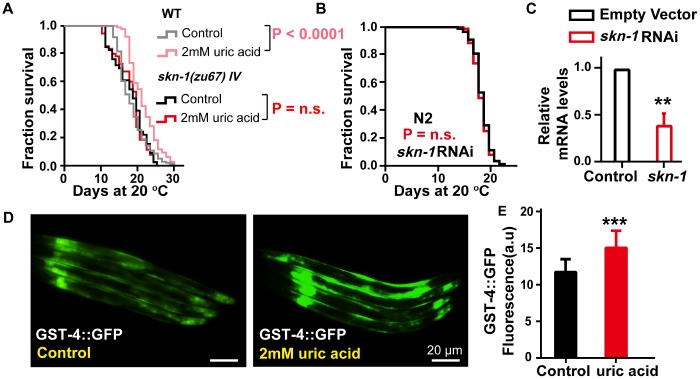
**The effect of uric acid on extending life span depends on its activation of transcription factor SKN-1.** (**A**) Life span analysis of the *skn-1(zu67)* mutant exposed to 2 mM uric acid and that of the untreated control. (**B**) Life span analysis of wild-type animals exposed to *skn-1* RNAi treated with 2 mM uric acid and that of the untreated control. The P value was calculated by the log-rank test, and the life span values of the replicated tests are listed in Supplementary Table 1. (**C**) *skn-1* RNA levels of animals after exposure to *skn-1* RNAi *E. coli* compared with the control (means ± SD, n = 3, ** P < 0.01, Student’s t test). (**D**, **E**) Image and quantitation of GFP fluorescence in the transgenic strain CL2166 (gst-4p::GFP) (means ± SD, n ≥ 30, *** P < 0.001, Student’s t test).

The antioxidant effect is mediated through the conserved oxidative and xenobiotic stress-response transcription factor SKN-1, an NRF2 ortholog that has remarkably conserved function relative to its mammalian counterpart [26]. Our results showed that the longevity-promoting effect of uric acid was completely lost in *skn-1(zu67)* mutants (Figure 3A). In line with the results from experiments on of the mutant, the prolongation of life span induced by uric acid was abrogated when s*kn-1* levels were knocked down by RNA interference (RNAi) in wild-type *C. elegans* (Figure 3B and 3C). Furthermore, we examined the expression of glutathione S-transferase 4 (GST-4), which is a target gene active in phase II detoxification that is regulated by SKN-1 in transgenic strain CL2166 [27]. We found that the CL2166 nematodes exposed to 2 mM uric acid showed significantly elevated fluorescence intensity in the assay compared with that of the untreated control (Figure 3D and 3E). These data illustrated that the antioxidant effect of uric acid also involved the activation of SKN-1.

### Uric acid extends the life span through the IIS signaling pathway

**Figure 4:**
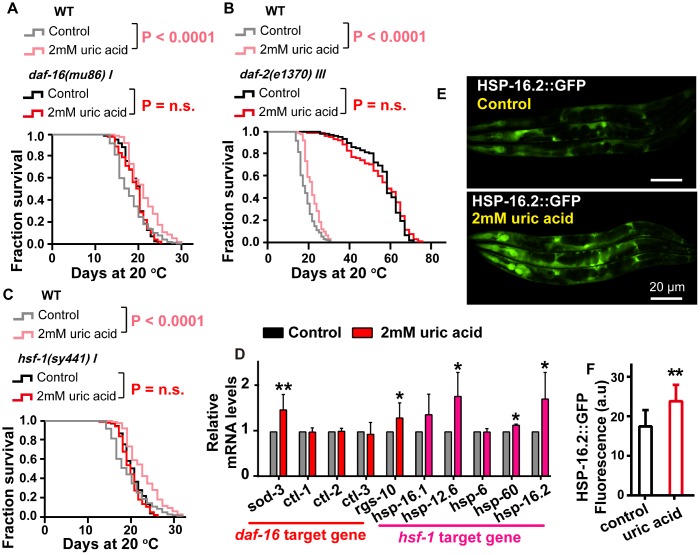
**The effect of uric acid on extending life span depends on its regulation of the IIS signaling pathway.** Survival analysis of (**A**) *daf-16 (mu86),* (**B**) *daf-2 (e1370),* (**C**) *hsf-1 (sy441)* mutant worms treated with 2 mM uric acid and of the untreated control (P value by log-rank test). The life span values of the repeat experiments are summarized in Supplementary Table 1. (**D**) QPCR analysis of the mRNA level of target genes *daf-16* and *hsf-1* when animals were exposed to 2 mM uric acid versus the control (means ± SD, n = 3, * P < 0.05, ** P < 0.01, Student’s t test). (**E**, **F**) Images and quantification of GFP fluorescence of transgenic strain CL2070 (hsp-16.2p::GFP). Data are the means ± SD, n ≥ 30, ** p < 0.01 (Student’s t test).

DAF-16, the *C. elegans* Forkhead box O transcription factor (FOXO) homolog, is a key factor that responds to different stresses, including oxidative stress [28]. To determine whether uric acid-induced life span expansion depends on DAF-16, we analyzed the survival of the *daf-16*-null mutant, *daf-16(mu86)* nematode after treatment with uric acid and found that uric acid failed to increase the life span of the *daf-16* mutant (Figure 4A), indicating that the longevity-promoting effects of uric acid are required for DAF-16 action. We also found that uric acid treatment increased the endogenous mRNA levels of *sod-3* and *rgs-10*, which are DAF-16/FOXO specific target genes [28] (Figure 4D). Consequently, our results suggest that the presence of uric acid may activate DAF-16.

DAF-16 is a considerably important downstream effector of the IIS pathway [29]. Considering that the uric acid-induced life span extension is mediated by DAF-16, we examined the role of the IIS pathway in uric acid-induced life span extension. Indeed, the specimens with the long-lived insulin-like receptor mutant *daf-2* had a similar mean and maximal life span when treated with either the uric acid or the vehicle (Figure 4B).

Heat-shock transcription factor (HSF-1), a stress response regulator, is a crucial longevity transcription factor known to act downstream of the IIS signaling pathway [20, 30]. To investigate whether the IIS signaling pathway is regulated by uric acid in an HSF-1-dependent manner, we examined the effect of 2 mM uric acid on the *hsf-1(sy441)* nematode. We found that, similar to that of the *daf-16* mutants, the life span of *hsf-1(sy441)* was not extended by uric acid (Figure 4C). In addition, the transcription levels of the target genes of HSF-1 (*hsp-12.6*, *hsp-16.1*, *hsp-16.2*, *hsp-6* and *hsp-60*) [31, 32] were significantly increased in the animals treated with 2 mM uric acid compared with the nontreatment control (Figure 4D). Furthermore, we also detected that the GFP fluorescence intensity of transgenic strains HSP-16.2::GFP was notably elevated, compared with that of the vehicle, when they were exposed to uric acid (Figure 4E and 4F). Collectively, the life span extension induced by uric acid might be mediated through its inhibition of the IIS signaling pathway, subsequently activating the downstream transcription factors DAF-16 and HSF-1.

### Uric acid extends the life span of *C. elegans* by regulating reproductive signaling pathway

**Figure 5:**
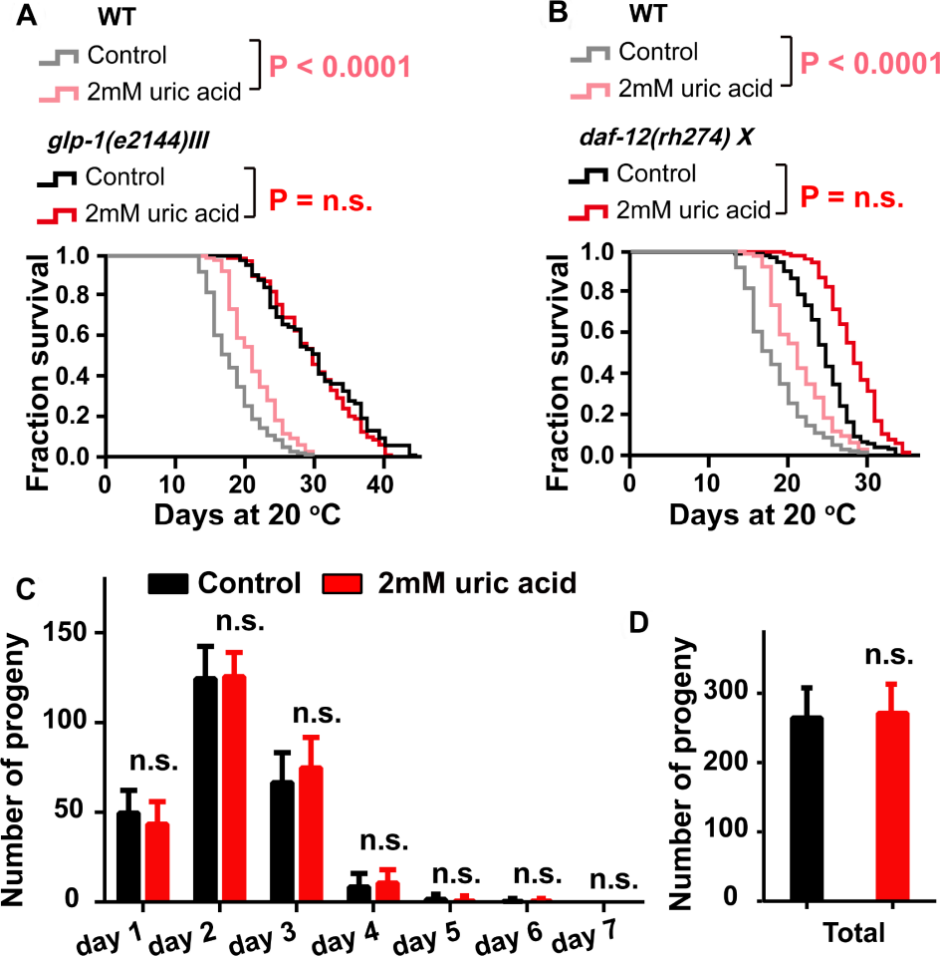
**The effect of uric acid on extending life span depends on its regulation of the reproductive signaling pathway.** Life span analysis for (**A**) *glp-1 (e2144)* and (**B**) *daf-12 (rh274)* mutant worms when treated with 2 mM uric acid versus control (P value by log-rank test). Detailed life span values are listed in Supplementary Table 1. (**C** and **D**) The number of daily progeny and the total number of progeny of wild-type N2 worms treated with 2 mM uric acid or vehicle (means ± SD, n ≥ 30, n.s.: no significant difference, Student’s t test).

The activation of DAF-16 by uric acid is reminiscent of the mechanism regulated in the reproductive signaling pathway. In *C. elegans*, removing the germline extended the life span by approximately 60% by activating DAF-16, at least in part [33, 34]. We tested the effect of uric acid on the long-lived *glp-1(e2144)* mutant, which has a germline loss due to failed germline proliferation when maintained at the nonpermissive temperature [35]. Our results showed that uric acid was unable to extend the life span of *glp-1* mutant worms (Figure 5A). Interestingly, we found that uric acid could further increase the life span of the *daf-12(rh274)* worms (Figure 5B)*.* DAF-12, a nuclear steroid receptor, is a crucial regulator downstream of the reproductive signaling pathway and mediates the life span regulation induced by germline loss in *C. elegans* [36, 37]. Consequently, our results illustrated that the longevity-promoting effect of uric acid is mediated through its regulation of reproductive signaling, which depends on DAF-16 but not DAF-12. Uric acid-induced prolongation depends on reproductive signaling pathways, which raises questions whether uric acid-induced prolongation is related to reproductive effects. However, no difference in the daily progeny production and total progeny were observed when animals treated with 2 mM uric acid compared with non-treated control (Figure 5C). One possible explanation is that uric acid only inhibits reproductive signaling pathways of *C. elegans*, but does not affect the reproductive capacity.

### Uric acid extends the life span through the mitochondrial-related signaling pathway

**Figure 6:**
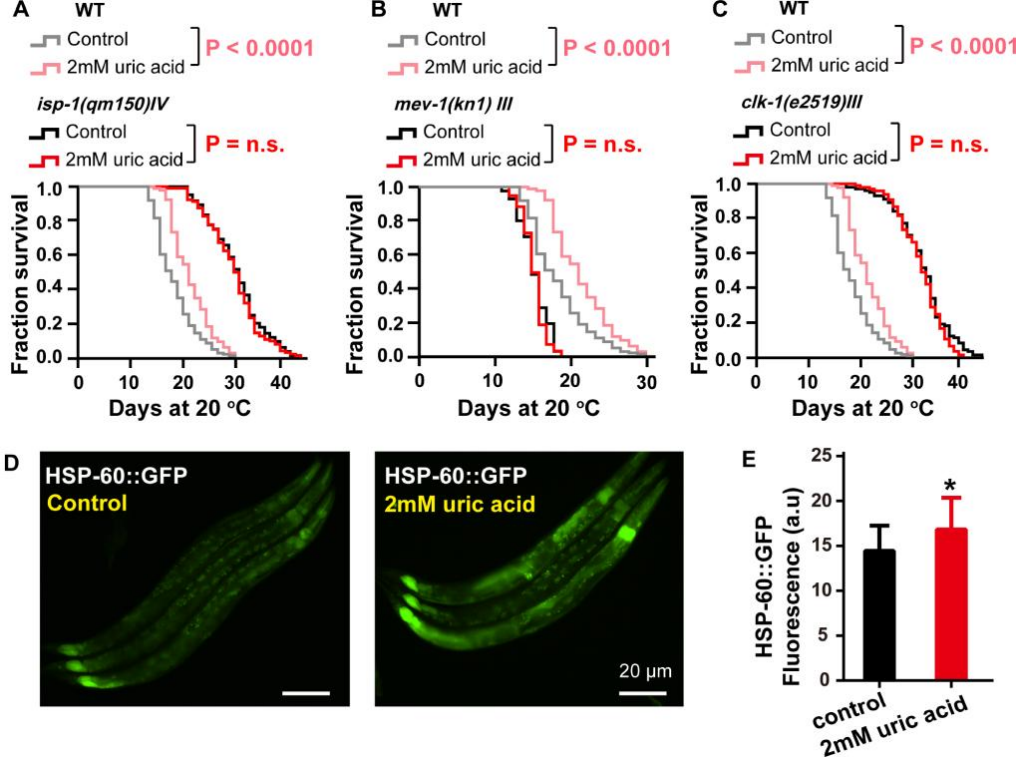
**The effect of uric acid on extending life span depends on mitochondrial function.** Survival analysis of (**A**) *isp-1 (qm150)*, (**B**) *mev-1 (kn1),* (**C**) *clk-1 (e2519)* mutant animals treated with 2 mM uric acid versus control (P value by log-rank test). Details on the life span values are presented in Supplementary Table 1. (**D**, **E**) Images and quantification of GFP fluorescence of transgenic strain SJ4058 (hsp-60p::GFP). Data are the means ± SD, n ≥ 30, * p < 0.05 (Student’s t test).

As mentioned before, the antioxidant activity of uric acid contributes to its effect on life span extension. Reactive oxygen species (ROS) are generated as a byproduct of normal metabolism and are thought to be produced mainly in mitochondria [38]. It has been confirmed that altering the expression of some subunits of the mitochondrial complexes could significantly increase the life span of *C. elegans* [39, 40]. These findings motivated us to question whether mitochondrial function plays an important role in uric acid-induced life extension. Our results demonstrated that uric acid could not extend the life span of either the long-lived mitochondrial dysfunctional mutants *isp-1* (the *isp-1* gene encodes the Rieske iron-sulfur protein of mitochondrial respiratory chain complex III) [41] (Figure 6A) and *clk-1* (an ortholog of human COQ7 (coenzyme Q7, hydroxylase)) [42] (Figure 6C), or the short-lived mitochondrial dysfunctional mutant *mev-1* (the *mev-1* gene encodes the cytochrome b large subunit (Cyt-1/ceSDHC) in complex II of the mitochondrial electron transport chain) [43] (Figure 6B).

It has been reported that mutations in the mitochondrial respiratory chain can trigger the mitochondrial unfolded protein response (UPR^mit^), which contributes to life span extension [44]. Because uric acid acts through the mitochondrial pathway, we next investigated the action of uric acid on the UPR^mit^. Similar to the results observed with the mitochondrial dysfunction mutants, the UPR^mit^ (GFP fluorescence intensity of HSP-60::GFP) was found to be increased notably when the animals were exposed to 2 mM uric acid compared with those animals that were untreated controls (Figure 6D and 6E); however, HSP-6 remained unchanged (Supplementary Figure 3). These results indicated that mitochondrial function and UPR^mit^ play important roles in the life span extension induced by uric acid.

## DISCUSSION

**Figure 7:**
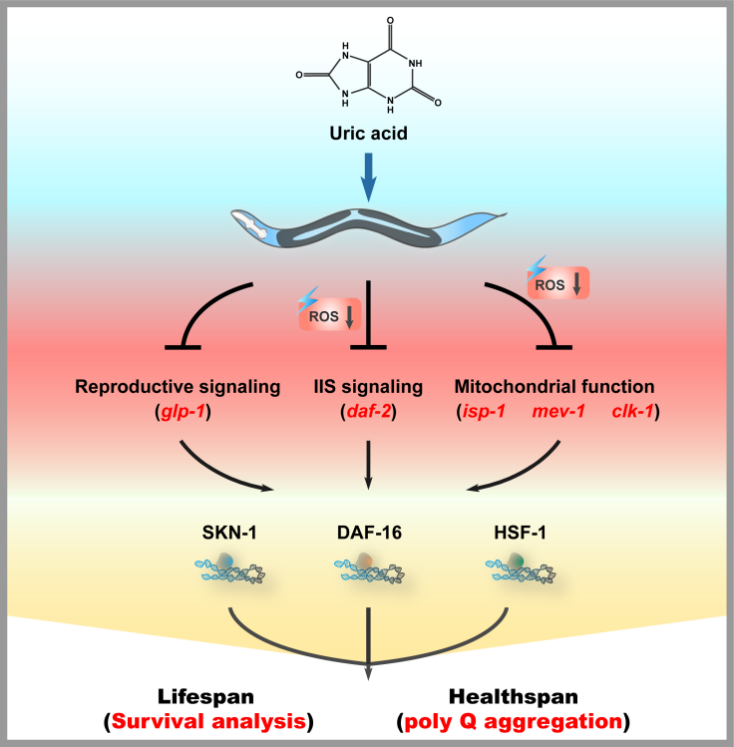
**Mechanisms of action of uric acid in *C. elegans*.**

ROS were generated from both endogenous and exogenous sources during aging. ROS have been increasingly recognized as a pivotal mediator of several oxidative stress responses, and an imbalance between ROS production and elimination has been considered a risk factor for aging and a number of age-related diseases. Some studies have reported a positive correlation between antioxidants and longevity. In this work, we investigated the impact of uric acid as an antioxidant on the health span and life span of nematode *C. elegans*. We confirmed that uric acid extends the life span in a remarkable way. We found that most aging pathways were required for uric acid-induced life span extension. Mutants defective for *daf-16* were unable to respond to uric acid. In addition, we found that uric acid-mediated life span extension depends on its regulation of the IIS signaling and reproductive signaling pathways. Moreover, we also found that *hsf-1* and mitochondrial dysfunction mutants (i.e., *isp-1*, *clk-1* and *mev-1*) were unresponsive to uric acid. Taken together, these observations are puzzling, as they illustrate that uric acid triggers many of the known longevity genes (Figure 7).

During aging, the resistance of *C. elegans* to external stimuli gradually decreased, and the accumulation of endogenous ROS gradually increased. Uric acid significantly improved oxidative stress resistance in *C. elegans* and effectively decreased ROS levels, suggesting that its antioxidative activity contributed to its longevity-promoting activity, at least in part. Indeed, we also observed that uric acid obviously increased the level of a number of antioxidant enzymes, including *sod-3* and *gst-4.* Here, *sod* genes encode superoxide dismutase, which converts superoxide, the primary form among the ROS generated in the mitochondria, into hydrogen peroxide. In addition, *gst* genes encode glutathione-S-transferase, which can detoxify products of oxidative stress. Unexpectedly, we found that uric acid failed to extend the life span of the *glp-1* mutant. A previous study had shown that the longevity of the *glp-1* mutant was associated with increased ROS levels in the body [35]. It seems to contradict the observed results that uric acid reduce ROS production. These observations also seem to indicate that the ability of uric acid to prolong life span depends not only on its antioxidant activity but also on its regulatory impact on other aging-related genes. Indeed, we also observed that life span extension induced by uric acid required for transcription factor DAF-16 and SKN-1. However, it is reported that DAF-16 and SKN-1 are activated under oxidative stress conditions [26, 28]. Uric acid, as an anti-oxidant, activated DAF-16 and SKN-1 to extend life span. One possible explanation is that several underlying molecular mechanisms activated by uric acid may be independent of its antioxidant, in which, uric acid inhibits reproductive signaling pathways, subsequently activates DAF-16 and SKN-1, in turn extends the life span of *C. elegans*.

Uric acid is a heterocyclic purine derivative that is the final oxidation product of purine metabolism. In our previous studies, we used metabolomics analysis and UPLC/MS and found that the aging of *C. elegans* was accompanied by a reduction in many intermediates of purine metabolism [45]. Consistent with previous studies, in the present study, we found that exogenously supplemented intermediates of purine metabolism, e.g., uric acid in food, significantly prolonged the life span of the nematodes. In support of our findings, other researchers have also found that supplementing the food of *C. elegans* with other purine metabolic intermediates, including allantoin [46] and xanthine [47], significantly extended the life span of worms. These results reveal that purine metabolic intermediates play an important role in the regulation of aging and that endogenous purine metabolites may be developed into potential strategies for the prevention and treatment of aging and age-related diseases.

In this work, our results revealed that the level of uric acid is positively correlated with life span. However, it is well known that, in humans, high levels of uric acid in plasma leads to a condition called hyperuricemia, while low levels are linked to a condition called hypouricemia. Additionally, hyperuricemia has been linked to a number of diseases and conditions, including gout, hypertension, cardiovascular disease, myocardial infarction, stroke, and renal disease [48–51], and hypouricemia has been associated with Parkinson’s disease and multiple sclerosis [52]. These studies of uric acid suggest that, due to the antioxidative activity of uric acid, higher concentrations of uric acid are generally beneficial compared with lower concentrations, but higher levels that result in crystal formation are detrimental. Therefore, in future research, our goal is to further clarify the molecular mechanism of uric acid regulation of life span and to determine the appropriate concentration that is beneficial to the health of the body.

## MATERIALS AND METHODS

### Chemicals, *C. elegans* strains, Maintenance, and life span assay

The *C. elegans* strains used in this work were obtained from the *Caenorhabditis* Genetic Center (CGC) (University of Minnesota, USA), which is supported by the NIH NCRR. Maintenance and synchronization of the nematodes, RNAi treatment and the life span assays were performed as previously described [53]. Life span assays were conducted using plates with 10 μM 5-fluoro-2’-deoxyuridine (FUdR, Sigma). The *skn-1* RNAi clone was obtained from the Ahringer library (Source BioScience, Nottingham, UK).

The following strains were used in this study: The wild-type *C. elegans* strain N2, AA89 *daf-12 (rh274)X*, CF1038 *daf-16 (mu86)I*, EU1 *skn-1 (zu67)IV*, PS3551 *hsf-1 (sy441)I*, VC199 *sir-2.1 (ok434)IV*, TK22 *mev-1 (kn1)III*, CB1370 *daf-2 (e1370)III*, CF1903 *glp-1 (e2144)III*, MQ887 *isp-1 (qm150)IV*, CB4876 *clk-1 (e2519)III*, CL2166 (dvIs19 [(pAF15) gst-4p::GFP::NLS])III, AM140 (rmIs132 [unc-54p::Q35::YFP]), CL2070(dvIs70 [hsp-16.2p::GFP + rol-6(su1006)]), SJ4058 (zcIs9 [hsp-60::GFP + lin-15(+)]), and SJ4100 (zcIs13[hsp-6::GFP]).

All compounds used in this work were purchased from Sigma-Aldrich (Munich, Germany). Uric acid and N-acetyl-cysteine (NAC) were dissolved in water. For dissolving uric acid, we used NaOH to adjust the PH of the solution to increase the solubility of uric acid in water. NGM plates containing these compounds were equilibrated overnight before use. Nematodes (wild-type N2 and the respective mutants) were raised on NGM plates for 2-3 generations without starvation before the initiation of experiments.

### Paraquat stress resistance assay

Oxidative stress resistance derived from paraquat was determined using a method, with minor modifications, previously described [54]. Young adult animals were transferred to fresh NGM plates containing 5 mM paraquat. The survival rate was determined as described for the life span analysis until all the worms died.

### Measurement of reactive oxygen species (ROS)

2′7′-Dichlorofluorescein diacetate (H2DCF-DA) was used to measure the levels of endogenous reactive oxygen species (ROS) [55]. L1 larvae were raised on NGM plates containing the respective compound until they reached the young adult stage, and then, the animals treated or untreated with compounds were transferred to plates with 10 mM H2DCF-DA and incubated for 1 h. Animals labeled with H2DCF-DA were placed on agar pads and imaged at 20× magnification using a Nikon Ti2-U fluorescence microscope. At least 30 worms were used for each experiment, and each assay was repeated at least twice. The fluorescence intensity of the images was analyzed by ImageJ software.

### Quantitative RT-PCR assay

Animals were synchronically grown on plates with or without uric acid at 20 °C as described for the life span assay. Total RNA was extracted using RNAiso Plus (TaKaRa), and then, 1 μg of total RNA was converted to cDNA following the manufacturer’s protocol. mRNA levels were quantified using SYBR Green Select Master Mix (Applied Biosystems) on a CFX96 real-time system (BioRad). Data were analyzed using the ΔΔCq method after normalization to the reference gene, *cdc-42* [56]. For every experiment, biological and technical triplicates were conducted. *P* values were calculated using two-tailed Student’s t test. The primers used in this publication are summarized in Supplementary Table 2, in Supporting Information.

### Fluorescence microscopic imaging

To measure the GFP fluorescence intensity of the *C. elegans*, populations of worms grown synchronically were anesthetized and arranged on an agar pad. For CL2166 (dvIs19 [Pgst-4p::GFP::NLS]), the L1 larvae were synchronically grown at plates with or without uric acid until they reached the young adult stage, and the GFP fluorescence of the worms was observed with a Nikon Ti2-U fluorescence microscope. The AM140 (rmIs132 [unc-54p::Q35::YFP]) worms were transferred during the spawning period to the plates, with or without compounds, for spawning; after 2 h, the worms were removed, the eggs continued hatching, and the larvae were growing until day 3 or day 4 of adulthood. The animals were placed on the agar pad for imaging. The CL2070 (dvIs70 [hsp-16.2p::GFP + rol-6(su1006)]), SJ4058 (zcIs9 [hsp-60::GFP + lin-15(+)]), SJ4100 (zcIs13[hsp-6::GFP]) transgenic strains L1 larvae were synchronically raised on plates with or without compounds until they reach the young adult stage, and then, animals were heated at 35 °C for 2 h to stimulate the expression of heat-shock protein. Images were taken and fluorescence quantification measured after the animals had recovered for 12 h at 20 °C. For all strains, the images were analyzed using ImageJ software. At least 30 worms were used for each experiment. *P* values were calculated using two-tailed Student’s t test.

### Fertility assay

Fertility assay was conducted as previously described [53]. The single L4 animals were transferred on NGM plates containing with or without uric acid, subsequently transferred to fresh plates every 24 h. Progeny were counted after hatching. For every experiment, more than 30 worms were used, and the experiments were conducted three times.

### Movement assay

Body movement assay was performed as previously described [53]. At least 60 young adults were transferred to fresh NGM plates with or without uric acid and maintained as described in the life span assay. The body movement was observed every day. When tapping plates, the animals moving in a continuous and coordinated sinusoidal were defined as fast movement, otherwise as a non-fast movement.

### Pharyngeal pumping rates

Pharyngeal pumping rates was counted as previously described [57]. At least 30 animals were transferred to fresh plates with or without compound, and maintained as described in the life span assay. Pharynx-pumping rate of worms were scored based on the grinder movement in the terminal bulb during a 60-s interval. The experiments were conducted three times, and for per experiment, each worm quantified in triplicates.

### Western blotting

Day 3 or day 4 animals maintained as described in fluorescence intensity analysis of AM140 were collected with M9 buffer. After three rounds of freezing and thaw, animals were lysed in RIPA buffer. And then RIPA samples were quantified with a BCA Protein Assay Kit, and boiled at 95 °C for 5 min. Protein were separated using SDS-PAGE and transferred to PVDF membrane. The membranes were blocked in milk, and then blotted with primary antibody against GFP (1:5000, ROCHE, 11814460001) or actin (1:5000, Sigma, A1978). The primary antibody was visualized using horseradish peroxidase-conjugated anti-mouse secondary antibody (1:2000) and ECL Western Blotting Substrate.

### Analysis of polyQ aggregates

Analysis of polyQ aggregates was performed as previously described [58]. Day 3 or day 4 worms were maintained as described in fluorescence intensity analysis of AM140. The number of polyQ aggregates were counted in individual animals at indicated days of adulthood. For every experiment, more than 30 worms at corresponding stages were used, and the experiments were conducted three times.

## Supplementary Material

Supplementary Figures

Supplementary Table 1

Supplementary Table 2

## References

[1] López-Otín C, Blasco MA, Partridge L, Serrano M, Kroemer G. The hallmarks of aging. Cell. 2013; 153:1194–217. 10.1016/j.cell.2013.05.039 23746838 PMC3836174

[2] Campisi J, Kapahi P, Lithgow GJ, Melov S, Newman JC, Verdin E. From discoveries in ageing research to therapeutics for healthy ageing. Nature. 2019; 571:183–92. 10.1038/s41586-019-1365-2 31292558 PMC7205183

[3] Balaban RS, Nemoto S, Finkel T. Mitochondria, oxidants, and aging. Cell. 2005; 120:483–95. 10.1016/j.cell.2005.02.001 15734681

[4] Harman D. Free radical theory of aging. Mutat Res. 1992; 275:257–66. 10.1016/0921-8734(92)90030-s 1383768

[5] Bokov A, Chaudhuri A, Richardson A. The role of oxidative damage and stress in aging. Mech Ageing Dev. 2004; 125:811–26. 10.1016/j.mad.2004.07.009 15541775

[6] Stadtman ER. Protein oxidation and aging. Science. 1992; 257:1220–24. 10.1126/science.1355616 1355616

[7] Dias V, Junn E, Mouradian MM. The role of oxidative stress in Parkinson’s disease. J Parkinsons Dis. 2013; 3:461–91. 10.3233/JPD-130230 24252804 PMC4135313

[8] Douglas PM, Dillin A. Protein homeostasis and aging in neurodegeneration. J Cell Biol. 2010; 190:719–29. 10.1083/jcb.201005144 20819932 PMC2935559

[9] Bonnefont-Rousselot D, Collin F. Melatonin: action as antioxidant and potential applications in human disease and aging. Toxicology. 2010; 278:55–67. 10.1016/j.tox.2010.04.008 20417677

[10] Dehghan E, Zhang Y, Saremi B, Yadavali S, Hakimi A, Dehghani M, Goodarzi M, Tu X, Robertson S, Lin R, Chudhuri A, Mirzaei H. Hydralazine induces stress resistance and extends C. elegans lifespan by activating the NRF2/SKN-1 signalling pathway. Nat Commun. 2017; 8:2223. 10.1038/s41467-017-02394-3 29263362 PMC5738364

[11] Davinelli S, Bertoglio JC, Polimeni A, Scapagnini G. Cytoprotective Polyphenols Against Chronological Skin Aging and Cutaneous Photodamage. Curr Pharm Des. 2018; 24:99–105. 10.2174/1381612823666171109102426 29119916

[12] Girgih AT, He R, Malomo S, Offengenden M, Wu JP, Aluko RE. Structural and functional characterization of hemp seed (Cannabis sativa L.) protein-derived antioxidant and antihypertensive peptides. J Funct Foods. 2014; 6:384–94. 10.1016/j.jff.2013.11.005

[13] Wang J, Hu S, Nie S, Yu Q, Xie M. Reviews on Mechanisms of In Vitro Antioxidant Activity of Polysaccharides. Oxid Med Cell Longev. 2016; 2016:5692852. 10.1155/2016/5692852 26682009 PMC4670676

[14] Hooper DC, Spitsin S, Kean RB, Champion JM, Dickson GM, Chaudhry I, Koprowski H. Uric acid, a natural scavenger of peroxynitrite, in experimental allergic encephalomyelitis and multiple sclerosis. Proc Natl Acad Sci USA. 1998; 95:675–80. 10.1073/pnas.95.2.675 9435251 PMC18479

[15] Hooper DC, Bagasra O, Marini JC, Zborek A, Ohnishi ST, Kean R, Champion JM, Sarker AB, Bobroski L, Farber JL, Akaike T, Maeda H, Koprowski H. Prevention of experimental allergic encephalomyelitis by targeting nitric oxide and peroxynitrite: implications for the treatment of multiple sclerosis. Proc Natl Acad Sci USA. 1997; 94:2528–33. 10.1073/pnas.94.6.2528 9122229 PMC20122

[16] Aliena-Valero A, López-Morales MA, Burguete MC, Castelló-Ruiz M, Jover-Mengual T, Hervás D, Torregrosa G, Leira EC, Chamorro Á, Salom JB. Emergent Uric Acid Treatment is Synergistic with Mechanical Recanalization in Improving Stroke Outcomes in Male and Female Rats. Neuroscience. 2018; 388:263–73. 10.1016/j.neuroscience.2018.07.045 30077000

[17] Amaro S, Jiménez-Altayó F, Chamorro Á. Uric acid therapy for vasculoprotection in acute ischemic stroke. Brain Circ. 2019; 5:55–61. 10.4103/bc.bc_1_19 31334357 PMC6611195

[18] Chigurupati S, Mughal MR, Chan SL, Arumugam TV, Baharani A, Tang SC, Yu QS, Holloway HW, Wheeler R, Poosala S, Greig NH, Mattson MP. A synthetic uric acid analog accelerates cutaneous wound healing in mice. PLoS One. 2010; 5:e10044. 10.1371/journal.pone.0010044 20386608 PMC2850366

[19] Cutler RG, Camandola S, Feldman NH, Yoon JS, Haran JB, Arguelles S, Mattson MP. Uric acid enhances longevity and endurance and protects the brain against ischemia. Neurobiol Aging. 2019; 75:159–68. 10.1016/j.neurobiolaging.2018.10.031 30576885 PMC6410356

[20] Lapierre LR, Hansen M. Lessons from C. elegans: signaling pathways for longevity. Trends Endocrinol Metab. 2012; 23:637–44. 10.1016/j.tem.2012.07.007 22939742 PMC3502657

[21] Harman D. Aging: a theory based on free radical and radiation chemistry. J Gerontol. 1956; 11:298–300. 10.1093/geronj/11.3.298 13332224

[22] Eruslanov E, Kusmartsev S. Identification of ROS using oxidized DCFDA and flow-cytometry. Methods Mol Biol. 2010; 594:57–72. 10.1007/978-1-60761-411-1_4 20072909

[23] Pole A, Dimri M, Dimri GP. Oxidative stress, cellular senescence and ageing. AIMS Mol Sci. 2016; 3:300–24. 10.3934/molsci.2016.3.300

[24] Barardo D, Thornton D, Thoppil H, Walsh M, Sharifi S, Ferreira S, Anžič A, Fernandes M, Monteiro P, Grum T, Cordeiro R, De-Souza EA, Budovsky A, et al. The DrugAge database of aging-related drugs. Aging Cell. 2017; 16:594–97. 10.1111/acel.12585 28299908 PMC5418190

[25] Lang S, Hilsabeck TA, Wilson KA, Sharma A, Bose N, Brackman DJ, Beck JN, Chen L, Watson MA, Killilea DW, Ho S, Kahn A, Giacomini K, et al. A conserved role of the insulin-like signaling pathway in diet-dependent uric acid pathologies in Drosophila melanogaster. PLoS Genet. 2019; 15:e1008318. 10.1371/journal.pgen.1008318 31415568 PMC6695094

[26] Tullet JM, Green JW, Au C, Benedetto A, Thompson MA, Clark E, Gilliat AF, Young A, Schmeisser K, Gems D. The SKN-1/Nrf2 transcription factor can protect against oxidative stress and increase lifespan in C. elegans by distinct mechanisms. Aging Cell. 2017; 16:1191–94. 10.1111/acel.12627 28612944 PMC5595692

[27] Ravichandran M, Priebe S, Grigolon G, Rozanov L, Groth M, Laube B, Guthke R, Platzer M, Zarse K, Ristow M. Impairing L-Threonine Catabolism Promotes Healthspan through Methylglyoxal-Mediated Proteohormesis. Cell Metab. 2018; 27:914–925.e5. 10.1016/j.cmet.2018.02.004 29551589

[28] Murphy CT, McCarroll SA, Bargmann CI, Fraser A, Kamath RS, Ahringer J, Li H, Kenyon C. Genes that act downstream of DAF-16 to influence the lifespan of Caenorhabditis elegans. Nature. 2003; 424:277–83. 10.1038/nature01789 12845331

[29] Ogg S, Paradis S, Gottlieb S, Patterson GI, Lee L, Tissenbaum HA, Ruvkun G. The Fork head transcription factor DAF-16 transduces insulin-like metabolic and longevity signals in C. elegans. Nature. 1997; 389:994–99. 10.1038/40194 9353126

[30] Seo K, Choi E, Lee D, Jeong DE, Jang SK, Lee SJ. Heat shock factor 1 mediates the longevity conferred by inhibition of TOR and insulin/IGF-1 signaling pathways in C. elegans. Aging Cell. 2013; 12:1073–81. 10.1111/acel.12140 23879233

[31] Chiang WC, Ching TT, Lee HC, Mousigian C, Hsu AL. HSF-1 regulators DDL-1/2 link insulin-like signaling to heat-shock responses and modulation of longevity. Cell. 2012; 148:322–34. 10.1016/j.cell.2011.12.019 22265419 PMC3615449

[32] Labbadia J, Brielmann RM, Neto MF, Lin YF, Haynes CM, Morimoto RI. Mitochondrial Stress Restores the Heat Shock Response and Prevents Proteostasis Collapse during Aging. Cell Rep. 2017; 21:1481–94. 10.1016/j.celrep.2017.10.038 29117555 PMC5726777

[33] Amrit FR, Steenkiste EM, Ratnappan R, Chen SW, McClendon TB, Kostka D, Yanowitz J, Olsen CP, Ghazi A. DAF-16 and TCER-1 Facilitate Adaptation to Germline Loss by Restoring Lipid Homeostasis and Repressing Reproductive Physiology in C. elegans. PLoS Genet. 2016; 12:e1005788. 10.1371/journal.pgen.1005788 26862916 PMC4749232

[34] Berman JR, Kenyon C. Germ-cell loss extends C. elegans life span through regulation of DAF-16 by kri-1 and lipophilic-hormone signaling. Cell. 2006; 124:1055–68. 10.1016/j.cell.2006.01.039 16530050

[35] Wei Y, Kenyon C. Roles for ROS and hydrogen sulfide in the longevity response to germline loss in Caenorhabditis elegans. Proc Natl Acad Sci USA. 2016; 113:E2832–41. 10.1073/pnas.1524727113 27140632 PMC4878494

[36] Motola DL, Cummins CL, Rottiers V, Sharma KK, Li T, Li Y, Suino-Powell K, Xu HE, Auchus RJ, Antebi A, Mangelsdorf DJ. Identification of ligands for DAF-12 that govern dauer formation and reproduction in C. elegans. Cell. 2006; 124:1209–23. 10.1016/j.cell.2006.01.037 16529801

[37] Antebi A. Regulation of longevity by the reproductive system. Exp Gerontol. 2013; 48:596–602. 10.1016/j.exger.2012.09.009 23063987 PMC3593982

[38] Weinberg F, Hamanaka R, Wheaton WW, Weinberg S, Joseph J, Lopez M, Kalyanaraman B, Mutlu GM, Budinger GR, Chandel NS. Mitochondrial metabolism and ROS generation are essential for Kras-mediated tumorigenicity. Proc Natl Acad Sci USA. 2010; 107:8788–93. 10.1073/pnas.1003428107 20421486 PMC2889315

[39] Ryu D, Mouchiroud L, Andreux PA, Katsyuba E, Moullan N, Nicolet-Dit-Félix AA, Williams EG, Jha P, Lo Sasso G, Huzard D, Aebischer P, Sandi C, Rinsch C, Auwerx J. Urolithin A induces mitophagy and prolongs lifespan in C. elegans and increases muscle function in rodents. Nat Med. 2016; 22:879–88. 10.1038/nm.4132 27400265

[40] Dillin A, Hsu AL, Arantes-Oliveira N, Lehrer-Graiwer J, Hsin H, Fraser AG, Kamath RS, Ahringer J, Kenyon C. Rates of behavior and aging specified by mitochondrial function during development. Science. 2002; 298:2398–401. 10.1126/science.1077780 12471266

[41] Rea SL, Ventura N, Johnson TE. Relationship between mitochondrial electron transport chain dysfunction, development, and life extension in Caenorhabditis elegans. PLoS Biol. 2007; 5:e259. 10.1371/journal.pbio.0050259 17914900 PMC1994989

[42] Larsen PL, Clarke CF. Extension of life-span in Caenorhabditis elegans by a diet lacking coenzyme Q. Science. 2002; 295:120–23. 10.1126/science.1064653 11778046

[43] Yanase S, Yasuda K, Ishii N. Adaptive responses to oxidative damage in three mutants of Caenorhabditis elegans (age-1, mev-1 and daf-16) that affect life span. Mech Ageing Dev. 2002; 123:1579–87. 10.1016/S0047-6374(02)00093-3 12470895

[44] Durieux J, Wolff S, Dillin A. The cell-non-autonomous nature of electron transport chain-mediated longevity. Cell. 2011; 144:79–91. 10.1016/j.cell.2010.12.016 21215371 PMC3062502

[45] Wan QL, Shi X, Liu J, Ding AJ, Pu YZ, Li Z, Wu GS, Luo HR. Metabolomic signature associated with reproduction-regulated aging in *Caenorhabditis elegans*. Aging (Albany NY). 2017; 9:447–74. 10.18632/aging.101170 28177875 PMC5361674

[46] Calvert S, Tacutu R, Sharifi S, Teixeira R, Ghosh P, de Magalhães JP. A network pharmacology approach reveals new candidate caloric restriction mimetics in C. elegans. Aging Cell. 2016; 15:256–66. 10.1111/acel.12432 26676933 PMC4783339

[47] Gioran A, Piazzesi A, Bertan F, Schroer J, Wischhof L, Nicotera P, Bano D. Multi-omics identify xanthine as a pro-survival metabolite for nematodes with mitochondrial dysfunction. EMBO J. 2019; 38:e99558. 10.15252/embj.201899558 30796049 PMC6418696

[48] Costa A, Igualá I, Bedini J, Quintó L, Conget I. Uric acid concentration in subjects at risk of type 2 diabetes mellitus: relationship to components of the metabolic syndrome. Metabolism. 2002; 51:372–75. 10.1053/meta.2002.30523 11887176

[49] Johnson RJ, Kang DH, Feig D, Kivlighn S, Kanellis J, Watanabe S, Tuttle KR, Rodriguez-Iturbe B, Herrera-Acosta J, Mazzali M. Is there a pathogenetic role for uric acid in hypertension and cardiovascular and renal disease? Hypertension. 2003; 41:1183–90. 10.1161/01.HYP.0000069700.62727.C5 12707287

[50] Feig DI, Kang DH, Johnson RJ. Uric acid and cardiovascular risk. N Engl J Med. 2008; 359:1811–21. 10.1056/NEJMra0800885 18946066 PMC2684330

[51] Messerli FH, Burnier M. Cardiovascular disease and uric acid: is the not-so-innocent bystander becoming a true culprit and does the US black box warning for febuxostat indicate that not all uric acid lowering is beneficial? Eur Heart J. 2019; 40:1787–89. 10.1093/eurheartj/ehz199 31173091

[52] Kutzing MK, Firestein BL. Altered uric acid levels and disease states. J Pharmacol Exp Ther. 2008; 324:1–7. 10.1124/jpet.107.129031 17890445

[53] Wan QL, Meng X, Fu X, Chen B, Yang J, Yang H, Zhou Q. Intermediate metabolites of the pyrimidine metabolism pathway extend the lifespan of *C. elegans* through regulating reproductive signals. Aging (Albany NY). 2019; 11:3993–4010. 10.18632/aging.102033 31232697 PMC6629003

[54] Zarse K, Schmeisser S, Groth M, Priebe S, Beuster G, Kuhlow D, Guthke R, Platzer M, Kahn CR, Ristow M. Impaired insulin/IGF1 signaling extends life span by promoting mitochondrial L-proline catabolism to induce a transient ROS signal. Cell Metab. 2012; 15:451–65. 10.1016/j.cmet.2012.02.013 22482728 PMC4844853

[55] Duangjan C, Rangsinth P, Gu X, Wink M, Tencomnao T. Lifespan Extending and Oxidative Stress Resistance Properties of a Leaf Extracts from *Anacardium occidentale* L. in *Caenorhabditis elegans*. Oxid Med Cell Longev. 2019; 2019:9012396. 10.1155/2019/9012396 31281595 PMC6589224

[56] Hoogewijs D, Houthoofd K, Matthijssens F, Vandesompele J, Vanfleteren JR. Selection and validation of a set of reliable reference genes for quantitative sod gene expression analysis in C. elegans. BMC Mol Biol. 2008; 9:9. 10.1186/1471-2199-9-9 18211699 PMC2254638

[57] Wan QL, Zheng SQ, Wu GS, Luo HR. Aspirin extends the lifespan of Caenorhabditis elegans via AMPK and DAF-16/FOXO in dietary restriction pathway. Exp Gerontol. 2013; 48:499–506. 10.1016/j.exger.2013.02.020 23485446

[58] Zhou Y, Wang X, Song M, He Z, Cui G, Peng G, Dieterich C, Antebi A, Jing N, Shen Y. A secreted microRNA disrupts autophagy in distinct tissues of Caenorhabditis elegans upon ageing. Nat Commun. 2019; 10:4827. 10.1038/s41467-019-12821-2 31645592 PMC6811558

