# Supplementary material for: Uric acid induces stress resistance and extends the life span through activating the stress response factor DAF-16/FOXO and SKN-1/NRF2
Source: Aging (Albany NY). 2020 Feb 12;12(3):2840–56. doi: 10.18632/aging.102781 (PMC7041755; doi:10.18632/aging.102781)
Supplement: Supplementary Figures [file aging-12-102781-s003..pdf]

SUPPLEMENTARY FIGURES

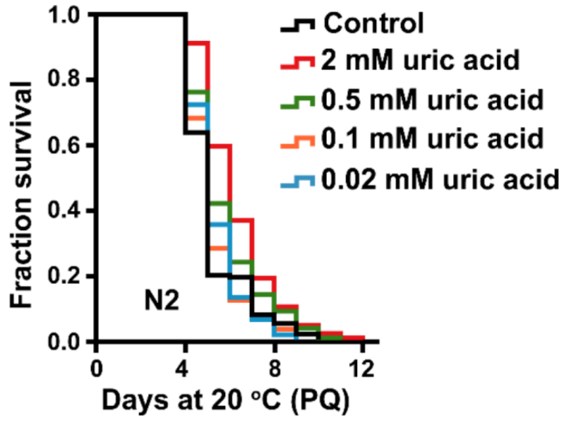

Supplementary Figure 1. Survival analysis of animals treated with different concentration of uric acid on paraquat and the untreated controls.

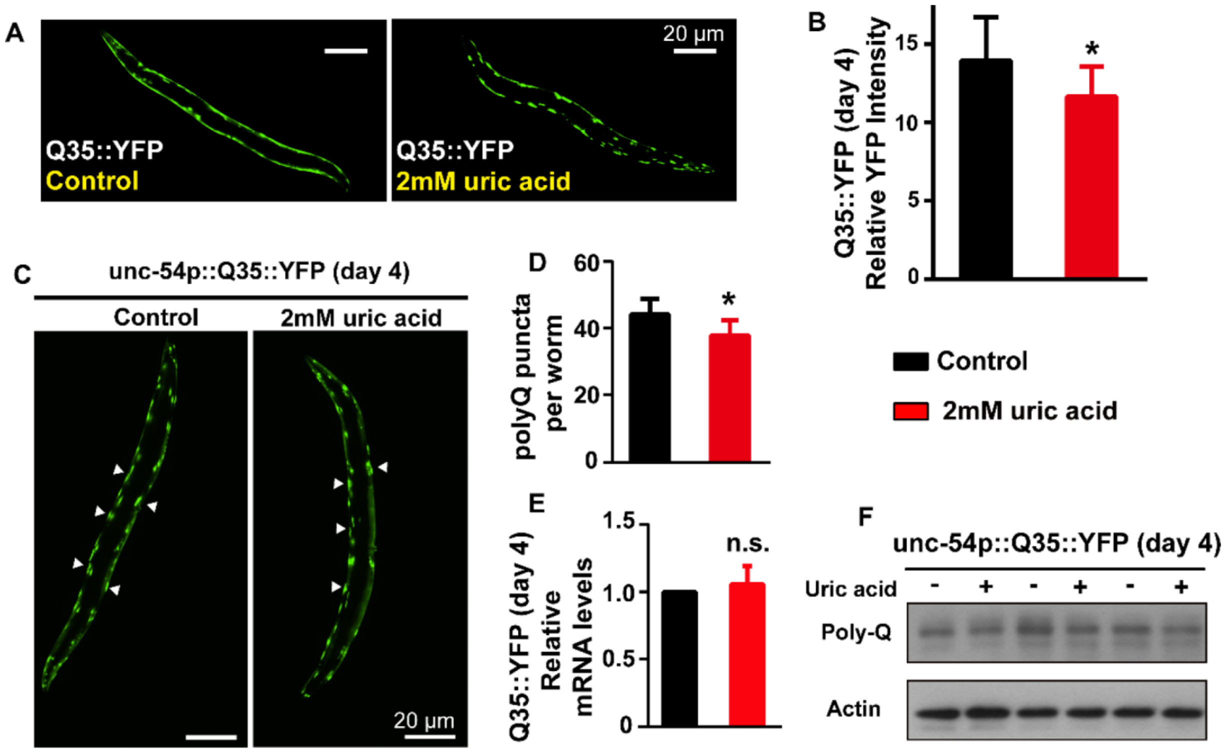

Supplementary Figure 2. The effect of uric acid on polyQ aggregation. (**A**, **B**) Image and quantitation of the fluorescent aggregation of AM140 at day 4 adulthood. Data are the means ± SD, n ≥ 30, * P < 0.05 (Student's t test). (**C**, **D**) Image and quantitation of the polyQ puncta aggregates of AM140 at day 4 adulthood (means ± SD, n ≥ 30, * p < 0.05 (Student's t test)) (**E**) mRNA level of Q35::YFP analysis when animals treated with or without 2 mM uric acid at day 4 adulthood (means ± SD, n =3, n.s.: no significant defference, Student's t test). (**F**) Western blot analysis of polyQ35::YFP in the presence and absence of 2 mM uric acid at day 4 of adulthood.

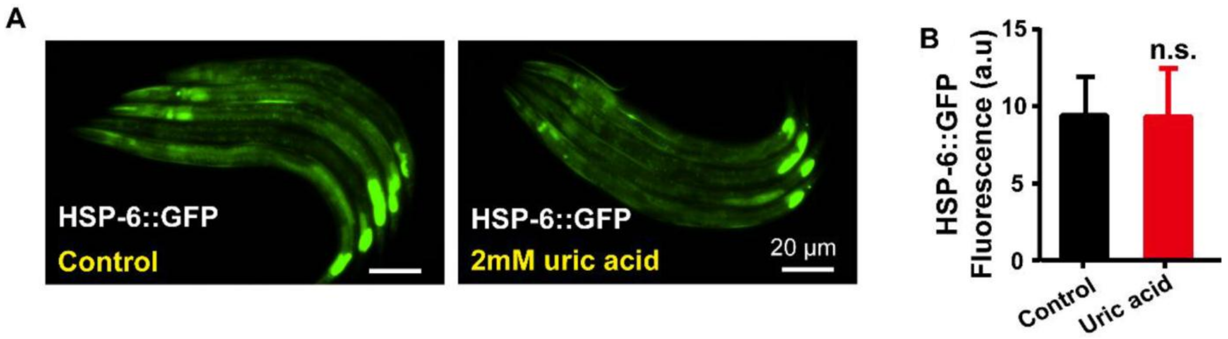

Supplementary Figure 3. The effect of uric acid on protein expression of HSP-6. (**A**, **B**) Image and quantitation of the fluorescent intensity of SJ4100 transgenic strain. Data are the means ± SD, n ≥ 30, P value was calculated by two-tailed Student's t test.
