# Supplementary material for: Uric acid induces stress resistance and extends the life span through activating the stress response factor DAF-16/FOXO and SKN-1/NRF2
Source: Aging (Albany NY). 2020 Feb 12;12(3):2840–56. doi: 10.18632/aging.102781 (PMC7041755; doi:10.18632/aging.102781)
Supplement: Supplementary Table 1 [file aging-12-102781-s001..docx]

**Supplementary Table 1. Survival analyses are conducted using the Kaplan-Meier method.**

| **Figure** | **Strains** | | **Treatments** | **Mean**  **Lifespan ± SEM**  **(days)** | ***P* value**  **VS**  **control** | **%**  **Change in mean lifespan** | **N** |
| --- | --- | --- | --- | --- | --- | --- | --- |
|  | **N2 (WT)** | |  |  |  |  |  |
| **Fig 1D, E & F** | EXP. 1 | | 20℃/ control | 19.478±0.296 |  |  | 136 |
|  | EXP. 1 | | 20℃/ 2 mM uric acid | 22.256±0.351 | <0.0001 | 14.26 | 90 |
|  | EXP. 1 | | 20℃/ 0.5 mM uric acid | 20.358±0.330 | 0.011 | 4.52 | 106 |
|  | EXP. 1 | | 20℃/ 0.1 mM uric acid | 20.650±0.361 | 0.009 | 6.02 | 117 |
|  | EXP. 1 | | 20℃/ 0.02 mM uric acid | 19.312±0.384 | 0.609 | # | 77 |
|  | EXP. 2 | | 20℃/ control | 21.198±0.230 |  |  | 101 |
|  | EXP. 2 | | 20℃/ 0.5 mM uric acid | 22.108±0.282 | 0.066 | # | 88 |
|  | EXP. 2 | | 20℃/ 0.1 mM uric acid | 23.239±0.363 | <0.0001 | 9.63 | 82 |
|  | EXP. 2 | | 20℃/ 0.02 mM uric acid | 22.073±0.283 | 0.016 | 4.13 | 137 |
|  | EXP. 3 | | 20℃/ control | 19.970±0.351 |  |  | 66 |
|  | EXP. 3 | | 20℃/ 2 mM uric acid | 22.475±0.467 | <0.0001 | 12.54 | 61 |
|  | EXP. 3 | | 20℃/ 0.5 mM uric acid | 20.961±0.328 | 0.015 | 4.96 | 77 |
|  | EXP. 3 | | 20℃/ 0.1 mM uric acid | 20.553±0.223 | 0.574 | # | 94 |
|  | EXP. 3 | | 20℃/ 0.02 mM uric acid | 20.756±0.383 | 0.084 | # | 90 |
|  | EXP. 4 | | 20℃/ control | 20.765±0.356 |  |  | 98 |
|  | EXP. 4 | | 20℃/ 2 mM uric acid | 24.022±0.395 | <0.0001 | 15.68 | 91 |
|  | EXP. 4 | | 20℃/ 10 mM uric acid | 23.758±0.354 | <0.0001 | 14.41 | 93 |
|  | EXP. 4 | | 20℃/ 50 mM uric acid | 21.441±0.394 | 0.054 | # | 111 |
|  | EXP. 4 | | 20℃/ 100 mM uric acid | 18.560±0.342 | <0.0001 | -10.62 | 114 |
|  | EXP. 5 | | 20℃/ control | 20.804±0.418 |  |  | 97 |
|  | EXP. 5 | | 20℃/ 2 mM uric acid | 23.183±0.394 | 0.030 | 11.43 | 104 |
|  | EXP. 5 | | 20℃/ 10 mM uric acid | 23.451±0.407 | 0.005 | 12.72 | 113 |
|  | EXP. 5 | | 20℃/ 50 mM uric acid | 21.920±0.427 | 0.956 | # | 88 |
|  | EXP. 5 | | 20℃/ 100 mM uric acid | 18.694±0.411 | <0.0001 | -10.14 | 72 |
|  | EXP. 6 | | 20℃/ control | 19.857±0.270 |  |  | 126 |
|  | EXP. 6 | | 20℃/ 2 mM uric acid | 22.524±0.366 | <0.0001 | 13.43 | 103 |
|  | EXP. 6 | | 20℃/ 10 mM uric acid | 22.324±0.322 | <0.0001 | 12.42 | 105 |
|  | EXP. 6 | | 20℃/ 50 mM uric acid | 20.034±0.260 | 0.465 | # | 148 |
|  | EXP. 6 | | 20℃/ 100 mM uric acid | 18.055±0.293 | 0.015 | -9.07 | 91 |
|  | EXP. 7 | | 20℃/ control | 20.72±0.253 |  |  | 133 |
|  | EXP. 7 | | 20℃/ 2 mM uric acid | 23.56±0.308 | <0.0001 | 13.71 | 150 |
|  | EXP. 7 | | 20℃/ 0.1 mM uric acid | 22.48±0.298 | 0.0399 | 8.49 | 138 |
|  | EXP. 7 | | 20℃/ 0.02 mM uric acid | 19.61±0.142 | 0.01 | -5.35 | 133 |
|  | EXP. 8 | | 20℃/ control | 22.554±0.441 |  |  | 61 |
|  | EXP. 8 | | 20℃/ 2 mM uric acid | 25.837±0.415 | <0.0001 | 14.56 | 80 |
|  | EXP. 9 | | 20℃/ control | 20.213±0.827 |  |  | 80 |
|  | EXP. 9 | | 20℃/ 2 mM uric acid | 23.376±0.809 | 0.001 | 15.65 | 83 |
|  |  | |  |  |  |  |  |
|  | **N2 (WT) with 5 mM PQ** | | |  |  |  |  |
| **Fig 1B**  **& Fig S1** | EXP. 1 | | 20℃/ control | 6.415±0.093 |  |  | 193 |
|  | EXP. 1 | | 20℃/ 2 mM uric acid | 7.333±0.105 | <0.0001 | 14.31 | 186 |
|  | EXP. 2 | | 20℃/ control | 6.545±0.085 |  |  | 299 |
|  | EXP. 2 | | 20℃/ 2 mM uric acid | 7.155±0.098 | <0.0001 | 9.32 | 309 |
|  | EXP. 3 | | 20℃/ control | 6.533±0.083 |  |  | 306 |
|  | EXP. 3 | | 20℃/ 2 mM uric acid | 7.137±0.094 | <0.0001 | 9.24 | 292 |
|  | EXP. 4 | | 20℃/control | 5.962±0.053 |  |  | 213 |
|  | EXP. 4 | | 20℃/ 2 mM uric acid | 6.574±0.082 | <0.0001 | 10.26 | 209 |
|  | EXP. 4 | | 20℃/ 0.5 mM uric acid | 5.995±0.064 | 0.700 | # | 215 |
|  | EXP. 4 | | 20℃/ 0.1 mM uric acid | 5.880±0.058 | 0.278 | # | 183 |
|  | EXP. 4 | | 20℃/ 0.02 mM uric acid | 5.912±0.058 | 0.522 | # | 194 |
|  | EXP. 5 | | 20℃/control | 6.029±0.072 |  |  | 140 |
|  | EXP. 5 | | 20℃/ 2 mM uric acid | 7.099±0.130 | <0.0001 | 17.75 | 142 |
|  | EXP. 5 | | 20℃/ 0.5 mM uric acid | 6.269±0.090 | 0.032 | 3.98 | 134 |
|  | EXP. 5 | | 20℃/ 0.1 mM uric acid | 6.086±0.096 | 0.600 | # | 105 |
|  | EXP. 5 | | 20℃/ 0.02 mM uric acid | 6.126±0.094 | 0.386 | # | 87 |
|  | EXP. 6 | | 20℃/control | 6.121±0.113 |  |  | 66 |
|  | EXP. 6 | | 20℃/ 2 mM uric acid | 7.382±0.175 | <0.0001 | 20.60 | 68 |
|  | EXP. 6 | | 20℃/ 0.5 mM uric acid | 6.697±0.175 | 0.006 | 9.41 | 66 |
|  | EXP. 6 | | 20℃/ 0.1 mM uric acid | 6.463±0.172 | 0.095 | # | 67 |
|  | EXP. 6 | | 20℃/ 0.02 mM uric acid | 6.164±0.098 | 0.791 | # | 73 |
|  | EXP. 7 | | 20℃/control | 6.170±0.105 |  |  | 182 |
|  | EXP. 7 | | 20℃/ 2 mM uric acid | 7.234±0.129 | <0.0001 | 17.24 | 158 |
|  | EXP. 7 | | 20℃/ 0.5 mM uric acid | 6.686±0.123 | 0.004 | 8.36 | 172 |
|  | EXP. 7 | | 20℃/ 0.1 mM uric acid | 6.178±0.097 | 0.790 | # | 157 |
|  | EXP. 7 | | 20℃/ 0.02 mM uric acid | .285±0.103 | 0.761 | # | 130 |
|  |  | |  |  |  |  |  |
|  | | ***skn-1 RNAi*** |  |  |  |  |  |
| **Fig 3B** | EXP. 1 | | 20℃/ control | 18.652±0.171 |  |  | 89 |
|  | EXP. 1 | | 20℃/ 2 mM uric acid | 18.477±0.166 | 0.430 | # | 88 |
|  | EXP. 2 | | 20℃/ control | 18.704±0.165 |  |  | 108 |
|  | EXP. 2 | | 20℃/ 2 mM uric acid | 18.990±0.159 | 0.287 | # | 103 |
|  | EXP. 3 | | 20℃/ control | 18.592±0.168 |  |  | 98 |
|  | EXP. 3 | | 20℃/ 2 mM uric acid | 18.888±0.178 | 0.227 | # | 89 |
|  |  | |  |  |  |  |  |
|  | ***skn-1 (zu67)IV*** | |  |  |  |  |  |
| **Fig 3A** | EXP. 1 | | 20℃/ control | 19.196±0.695 |  |  | 46 |
|  | EXP. 1 | | 20℃/ 2 mM uric acid | 19.255±0.598 | 0.747 | # | 55 |
|  | EXP. 2 | | 20℃/ control | 23.108±0.465 |  |  | 65 |
|  | EXP. 2 | | 20℃/ 2 mM uric acid | 22.111±0.506 | 0.077 | # | 63 |
|  | EXP. 3 | | 20℃/ control | 20.429±0.452 |  |  | 56 |
|  | EXP. 3 | | 20℃/ 2 mM uric acid | 20.228±0.574 | 0.616 | # | 57 |
|  |  | |  |  |  |  |  |
|  | ***daf-16 (mu86)I*** | |  |  |  |  |  |
| **Fig 4A** | EXP. 1 | | 20℃/ control | 20.545±0.435 |  |  | 77 |
|  | EXP. 1 | | 20℃/ 2 mM uric acid | 21.162±0.411 | 0.546 | # | 68 |
|  | EXP. 2 | | 20℃/ control | 19.743±0.353 |  |  | 101 |
|  | EXP. 2 | | 20℃/ 2 mM uric acid | 19.989±0.328 | 0.831 | # | 87 |
|  | EXP. 3 | | 20℃/ control | 21.155±0.321 |  |  | 103 |
|  | EXP. 3 | | 20℃/ 2 mM uric acid | 20.326±0.360 | 0.098 | # | 92 |
|  |  | |  |  |  |  |  |
|  | ***daf-2(e1370) III*** | |  |  |  |  |  |
| **Fig 4B** | EXP. 1 | | 20℃/ control | 52.611±0.922 |  |  | 108 |
|  | EXP. 1 | | 20℃/ 2 mM uric acid | 51.410±1.62 | 0.371 | # | 105 |
|  | EXP. 2 | | 20℃/ control | 53.716±0.938 |  |  | 95 |
|  | EXP. 2 | | 20℃/ 2 mM uric acid | 55.419±0.830 | 0.365 | # | 86 |
|  | EXP. 3 | | 20℃/ control | 53.883±0.801 |  |  | 111 |
|  | EXP. 3 | | 20℃/ 2 mM uric acid | 56.326±0.708 | 0.062 | # | 92 |
|  |  | |  |  |  |  |  |
|  | ***glp-1 (e21444)III*** | |  |  |  |  |  |
| **Fig 5A** | EXP. 1 | | 20℃/ control | 34.744±0.876 |  |  | 82 |
|  | EXP. 1 | | 20℃/ 2 mM uric acid | 34.218±0.769 | 0.274 | # | 78 |
|  | EXP. 2 | | 20℃/ control | 32.680±0.600 |  |  | 97 |
|  | EXP. 2 | | 20℃/ 2 mM uric acid | 32.413±0.630 | 0.441 | # | 75 |
|  | EXP. 3 | | 20℃/ control | 33.582±0.548 |  |  | 91 |
|  | EXP. 3 | | 20℃/ 2 mM uric acid | 33.394±0.574 | 0.502 | # | 66 |
|  |  | |  |  |  |  |  |
|  | ***daf-12 (rh274)X*** | |  |  |  |  |  |
| **Fig 5B** | EXP. 1 | | 20℃/ control | 25.395±0.314 |  |  | 114 |
|  | EXP. 1 | | 20℃/ 2 mM uric acid | 28.081±0.398 | <0.0001 | 10.57 | 99 |
|  | EXP. 2 | | 20℃/ control | 26.591±0.324 |  |  | 110 |
|  | EXP. 2 | | 20℃/ 2 mM uric acid | 29.964±0.375 | <0.0001 | 12.68 | 88 |
|  | EXP. 3 | | 20℃/ control | 27.841±0.381 |  |  | 113 |
|  | EXP. 3 | | 20℃/ 2 mM uric acid | 32.000±0.360 | <0.0001 | 14.93 | 109 |
|  |  | |  |  |  |  |  |
|  | ***hsf-1 (sy441)I*** | |  |  |  |  |  |
| **Fig 4C** | EXP. 1 | | 20℃/ control | 20.318±0.263 |  |  | 107 |
|  | EXP. 1 | | 20℃/ 2 mM uric acid | 20.309±0.260 | 0.968 | # | 110 |
|  | EXP. 2 | | 20℃/ control | 20.157±0.274 |  |  | 108 |
|  | EXP. 2 | | 20℃/ 2 mM uric acid | 19.347±0.272 | 0.067 | # | 124 |
|  | EXP. 3 | | 20℃/ control | 19.142±0.276 |  |  | 106 |
|  | EXP. 3 | | 20℃/ 2 mM uric acid | 19.635±0.275 | 0.276 | # | 115 |
|  |  | |  |  |  |  |  |
|  | ***isp-1 (qm150)IV*** | |  |  |  |  |  |
| **Fig 6A** | EXP. 1 | | 20℃/ control | 29.971±0.517 |  |  | 104 |
|  | EXP. 1 | | 20℃/ 2 mM uric acid | 30.482±0.582 | 0.535 | # | 85 |
|  | EXP. 2 | | 20℃/ control | 34.919±0.730 |  |  | 86 |
|  | EXP. 2 | | 20℃/ 2 mM uric acid | 33.925±0.616 | 0.149 | # | 106 |
|  | EXP. 3 | | 20℃/ control | 27.524±0.349 |  |  | 103 |
|  | EXP. 3 | | 20℃/ 2 mM uric acid | 27.448±0.370 | 0.923 | # | 105 |
|  |  | |  |  |  |  |  |
|  | ***mev-1 (kn1)III*** | |  |  |  |  |  |
| **Fig 6B** | EXP. 1 | | 20℃/ control | 14.765±0.207 |  |  | 81 |
|  | EXP. 1 | | 20℃/ 2 mM uric acid | 14.674±0.172 | 0.404 | # | 86 |
|  | EXP. 2 | | 20℃/ control | 15.325±0.175 |  |  | 77 |
|  | EXP. 2 | | 20℃/ 2 mM uric acid | 15.388±0.210 | 0.350 | # | 85 |
|  | EXP. 3 | | 20℃/ control | 14.758±0.216 |  |  | 91 |
|  | EXP. 3 | | 20℃/ 2 mM uric acid | 14.535±0.194 | 0.380 | # | 99 |
|  |  | |  |  |  |  |  |
|  | ***clk-1 (e2519)III*** | |  |  |  |  |  |
| **Fig 6C** | EXP. 1 | | 20℃/ control | 36.259±0.719 |  |  | 85 |
|  | EXP. 1 | | 20℃/ 2 mM uric acid | 37.657±0.645 | 0.061 | # | 99 |
|  | EXP. 2 | | 20℃/ control | 34.847±0.479 |  |  | 98 |
|  | EXP. 2 | | 20℃/ 2 mM uric acid | 35.198±0.574 | 0.156 | # | 101 |
|  | EXP. 3 | | 20℃/ control | 35.981±0.578 |  |  | 107 |
|  | EXP. 3 | | 20℃/ 2 mM uric acid | 36.279±0.589 | 0.985 | # | 86 |

*P* values were calculated by log-rank test for individual experiments. All statistical were performed using SPSS package. “N” displayed the number of dead worms. “#”: no calculate (because p > 0.05).
