# Supplementary material for: Uric acid induces stress resistance and extends the life span through activating the stress response factor DAF-16/FOXO and SKN-1/NRF2
Source: Aging (Albany NY). 2020 Feb 12;12(3):2840–56. doi: 10.18632/aging.102781 (PMC7041755; doi:10.18632/aging.102781)
Supplement: Supplementary Table 2 [file aging-12-102781-s002..pdf]

Supplementary Table 2. Sequences of PCR primers (qPCR).

Primer sequences used for quantitative PCR (5′→3′):		
**Gene**	**Forward primer sequence**	**Reverse primer sequence**
cdc-42	CTGCTGGACAGGAAGATTACG	CTCGGACATTCTCGAATGAAG
sod-3	AGCATCATGCCACCTACGTGA	AGCATCATGCCACCTACGTGA
skn-1	TGGAGTGTCGTCCATATTCATCT	TGAGGTGTTGGACGATGGTG
ctl-1	GACGTATCCAAAACCCCAAGTG	TTGGCATGAACGACACGCTC
ctl-2	TTCCGATCGAGGACTCCCAG	CTTCACTCCTTGAGTTGGCTTG
ctl-3	CCCACATGGTCAATCTAACGGT	GGAGCTCCATTGGATGTGGT
rgs-10	TAGTGATTCTGGAGCGTGTTG	TGGACGGAAGTTCATTAGAGC
hsp-16.1	GTCACTTTACCACTATTTCCGTCCAGCTCAACGTTC	CAACGGGCGCTTGCTGAATTGGAATAGATCTTCC
hsp-16.2	CTGCAGAATCTCTCCATCTGAGTC	AGATTCGAAGCAACTGCACC
hsp-12.6	GTGATGGCTGACGAAGGAAC	GGGAGGAAGTTATGGGCTTC
hsp-6	GGATGCTGGACAAATCTCTG	ACAGCGATGATCTTATCTCCA
hsp-60	AAGGATATGGGAATTGCGACGGGA	TGTGCTCGATTCGCTTCTCGATCT
YFP-F	TATATCATGGCCGACAAGCA	GTTGTGGCGGATCTTGAAGT
